# Autoimmunity Following Allogeneic Hematopoietic Stem Cell Transplantation

**DOI:** 10.3389/fimmu.2020.02017

**Published:** 2020-08-25

**Authors:** Nataliya Prokopenko Buxbaum, Steven Z. Pavletic

**Affiliations:** ^1^Experimental Transplantation and Immunotherapy Branch, Center for Cancer Research, National Cancer Institute, National Institutes of Health, Bethesda, MD, United States; ^2^Immune Deficiency Cellular Therapy Program, Center for Cancer Research, National Cancer Institute, National Institutes of Health, Bethesda, MD, United States

**Keywords:** autoimmunity, alloimmunity, hematopoietic stem cell transplantation, allogeneic, immune reconstitution, non-hematologic, autoimmune hemolytic anemia, autoimmune cytopenia

## Abstract

Autoimmune manifestations after allogeneic hematopoietic stem cell transplantation (AHSCT) are rare and poorly understood due to the complex interplay between the reconstituting immune system and transplant-associated factors. While autoimmune manifestations following AHSCT have been observed in children with graft-versus-host disease (GvHD), an alloimmune process, they are distinct from the latter in that they are generally restricted to the hematopoietic compartment, i.e., autoimmune hemolytic anemia, thrombocytopenia, and/or neutropenia. Autoimmune cytopenias in the setting of ASHCT represent a donor against donor immune reaction. Non-hematologic autoimmune conditions in the post-AHSCT setting have been described and do not currently fall under the GvHD diagnostic criteria, but could represent alloimmunity since they arise from the donor immune attack on the antigens that are shared by the donor and host in the thyroid, peripheral and central nervous systems, integument, liver, and kidney. As in the non-transplant setting, autoimmune conditions are primarily antibody mediated. In this article we review the incidence, risk factors, potential pathophysiology, treatment, and prognosis of hematologic and non-hematologic autoimmune manifestations in children after AHSCT.

## Introduction

Allogeneic hematopoietic stem cell transplantation (AHSCT) has the potential to cure refractory hematopoietic malignancies as well as acquired and inherited non-malignant immune diseases, hemoglobinopathies, and inherited metabolic disorders. For inherited disorders, emerging genetic therapies may offer an alternative (1, 2) while immunotherapeutic approaches, including AHSCT, will likely continue to be widely used for malignant diseases. Children are more likely to experience long term survival after AHSCT, but are also susceptible to harmful effects of AHSCT on growth and development of many organ systems (3). As more children undergo ASHCT, identification of biological risks that are unique to this population, and the underlying biological processes is needed.

Reconstitution of the adaptive immune system following AHSCT is primarily mediated through peripheral non-thymic expansion of donor-derived T cells in the host (4, 5). Even in children, thymic output is significantly reduced in the immediate post-HSCT period due to transplant-related insults. Thus, peripheral (non-thymic) immune tolerance mechanisms appear to be critical during this time of immune recovery and for the emergence of both alloimmune and autoimmune complications of AHSCT. Alloimmunity stems from the donor recognition of host and can be detrimental when it manifests as graft-versus-host disease (GvHD) due to the resultant attack on the recipient tissues (6) or beneficial when directed against the malignant cells, i.e., the graft-versus-leukemia (GVL) effect. GvHD is a common HSCT complication that has acute and chronic forms. Both have well-characterized clinicopathologic features involving the gastrointestinal tract, liver and skin, with additional organ involvement in chronic GvHD (7). Chronic GvHD (cGvHD) typically affects tissues that form the physical and immune barrier between the host and potential infectious pathogens and thus are enriched with immune cells, i.e., skin, eyes, pulmonary tract, mouth, gastrointestinal tract, and genital tract. Autoimmunity after HSCT affects tissues that are often targeted by idiopathic autoimmune diseases (AD).

Outside of the AHSCT setting the pathophysiology of autoimmunity is multifactorial and the exact timing and type of the inciting event is usually unknown. In the AHSCT setting, the timing of the autoimmunity initiating cascade starts with the donor cell infusion. AHSCT adds a number of potentially detrimental effects that can skew the reconstituting immune system toward AD and represents a unique clinical model for AD research (8, 9). Whether GvHD and AD after HSCT have shared pathophysiology is an active research question. Both are driven by donor immune reaction, in the former the targets are host, while AD is directed against the donor hematopoietic compartment, or non-hematopoietic targets that are common to the donor and host. Children experience lower rates of GvHD (3) but those that undergo AHSCT for non-malignant indications appear to have a higher risk of hematologic autoimmune manifestations (9). Despite non-hematologic autoimmune-like manifestations being less frequent in children than adults they too have a higher incidence in the setting of non-malignant AHSCT (10). Although isolated ADs following AHSCT are rare they are observed in higher frequency in patients with GvHD (8, 9).

A recent comprehensive review of the literature on hematologic AD in children identified non-malignant indication for AHSCT, the use of unrelated transplant donor, omission of total body irradiation in the conditioning regimen, presence of cGvHD, and the use of peripheral or cord blood stem cell grafts as significant risk factors (9). These provide important clues about potential pathophysiology of AD. AD after AHSCT is characterized by impaired immune reconstitution that may stem from either incomplete lymphodepletion prior to HSCT, possibly leaving partially intact the antigen presenting compartment, or permitting donor B cell expansion concomitant with significant T cell depletion of the graft and/or peri-transplant use of immunosuppression that preferentially suppresses donor T cell reconstitution. As a result, an imbalance in T and B cell immunity may lead to an impaired peripheral tolerance, facilitating immune dysregulation that allows emergence of autoimmunity after AHSCT (8, 9, 11, 12). In hematological AD after AHSCT, the reaction direction is most consistent with donor immune recognition of donor antigens. In cases of non-hematologic AD after AHSCT, donor immune recognition of shared donor-host antigens is likely, but residual tissue resident antigen presenting cells may be of host origin even when full donor chimerism is confirmed in circulating immune cells. Thus, the potential for non-hematologic AD to be of host against host direction cannot be eliminated at the present time. Future studies may enable delineation of immune cell chimerism in tissue and thus may provide clarity on the ontogeny of the immune reaction in non-hematologic AD after AHSCT.

The goal of this article is to comprehensively review hematologic and non-hematologic AD after AHSCT in children, summarized in Table 1. Furthermore, features of adult AD following HSCT and corresponding ADs observed outside of the HSCT setting are described with the aim of improving the combined understanding of the underlying biology, risk factors, and identifying potential interventions or changes to existing HSCT platforms that may need to be implemented.

**TABLE 1:** Incidence, risk factors, associated clinical features, and proposed mechanisms for autoimmune disease after AHSCT.

Disease	Incidence	Risk factors and clinical features	Proposed mechanism
Autoimmune cytopenia/s, including AIHA, ITP, Evans syndrome, AIN, and tri-lineage autoimmune cytopenia	Rare, but common in subsets of pediatric ASHCT compared to adult recipients	Non-malignant transplant indication	Skewing of immune reconstitution toward unregulated B cell proliferation and auto-antibody production due to impaired peripheral tolerance in the absence or reduced function of T cells
		Unrelated donor	
		Lack of TBI	
		cGvHD	
		peripheral or UCB stem cell source	
		additional risk factors in adult HSCT: T cell depleted grafts, ATG and alemtuzumab in the peri-transplant setting, GvHD	
Autoimmune thyroid disease, including Hashimoto thyroiditis and Graves’ disease	Rare, except for one pediatric study reporting 25% rate	Non-malignant transplant indication	Unchecked autoantibody production against thyroid antigens In adults, transmission of autoantibody in the graft has been described
		T cell depleted graft and/or ATG or alemtuzumab peri-transplant	
		Lack of TBI	
		Immune recovery (albeit dysfunctional)	
Guillain-Barre syndrome	Rare, 10 pediatric cases described	Malignant indication for AHSCT	Potentiation of Ara-C neurotoxicity Possible molecular mimicry of PNS antigens by viral antigens
		Associated with infection or viral reactivation	
		Antecedent use of high dose Ara-C (practice discontinued after this association was identified)	
Myasthenia Gravis	Exceedingly rare, 2 pediatric cases reported	Non-malignant transplant indication	Unchecked autoantibody production against acetylcholine receptor
		Acute and chronic GvHD	
		Manifested upon tapering of immunosuppression	
		Generalized severe presentation	
		No association with thymoma	
Transverse myelitis	Exceedingly rare, 1 pediatric case and several adult cases	Non-malignant transplant indication	Unchecked inflammatory milieu
		Unrelated donor	
		Lack of TBI	
		Peri-transplant use of alemtuzumab	
Other CNS manifestations, including vasculitis, white matter lesions and atrophy	Exceedingly rare, in children and adult recipients	Unrelated donor	Lymphocytic infiltration of CNS vasculature or parenchyma by immune cells of donor origin
		Manifested upon tapering of immunosuppression	
Vitiligo	Exceedingly rare, 8 pediatric cases reported, similar incidence in adult recipients	One pediatric series with non-malignant indication for AHSCT and no GvHD	Unchecked autoantibody production against melanocytes
		Another subset of patients with a malignant indication for HSCT and a GvHD association	
Autoimmune hepatitis	Exceedingly rare, 2 pediatric and one adult case described	Possibly associated with GvHD	Portal eosinophilia and plasma cell infiltration of donor origin
		Responded to immunosuppression	
Rheumatologic diseases, including arthritis, spondyloarthropathy, vasculitis, phospholipid antibody syndrome	Exceedingly rare in children with 2 possible young adult cases reported, more common in adults undergoing AHSCT for autoimmune disease	Both pediatric patients received AHSCT for a malignant indication	Predisposition toward autoimmunity that may be potentiated by ASHCT

## Hematopoietic Autoimmune Manifestations Following AHSCT

### Incidence

The most common autoimmune manifestations following AHSCT in pediatric and adult recipients are hematologic, i.e., autoimmune cytopenias (AICs) (8, 9). AICs are classified based on the affected lineage/s and include autoimmune hemolytic anemia (AIHA), immune thrombocytopenic purpura (ITP), Evans syndrome (AIHA and ITP), autoimmune neutropenia (AIN), and tri-lineage autoimmune cytopenia (AIHA with ITP and AIN) (12–14). While AIHA is the most commonly diagnosed AIC following AHSCT, accurate reporting of ITP in this setting is challenging because there are several alternate potential transplant related causes of thrombocytopenia that have to be excluded prior to making the diagnosis and laboratory confirmation of AIHA is more readily obtained compared with detection of anti-platelet antibodies, which are not uniformly observed in ITP (8, 9, 14). Of note, in the setting of AHSCT for acquired aplastic anemia, ITP incidence reportedly exceeds that of AIHA (14), and is the same as that of AIHA in autologous HSCT for ADs (15). Meanwhile, the incidence of AIHA in the general population is lower than in the post-AHSCT setting and far less common in children compared to adults (16, 17).

Despite AICs being rare following AHSCT with an estimated incidence of ∼3% in adults (14, 18–26) and ∼5% percent in children (9, 13, 26–31), they occur with much greater frequency in certain AHSCT settings. The highest AIC rates, over 50%, were reported in very young infants that received unrelated cord blood (UCB) grafts for inherited metabolic disorders (12) and those who received AHSCT for Wiscott-Aldrich syndrome (WAS) (32), with antithymocyte globulin (ATG)-containing conditioning used in both studies. Several additional case series that demonstrated higher than average AIC incidence of 20–35% (10, 11, 33) also involved children undergoing AHSCT for non-malignant indications following intense lymphodepletion. One such study reported a combined 50% rate of hematologic and non-hematologic AD in children following AHSCT for chronic granulomatous disease (CGD) following conditioning that included alemtuzumab (10).

### Risk Factors

The following significant risk factors for the development of AIC in children undergoing HSCT have been recently confirmed: non-malignant primary diagnosis, the use of an unrelated donor, lack of total body irradiation (TBI) in the conditioning regimen, chronic GvHD, and the use of peripheral or UCB stem cell source (9). Similar risk factors have been identified in the adult AHSCT literature with the strongest association seen between AIC and the use of unrelated donors, T cell depleted grafts, conditioning regimens that include ATG and alemtuzumab, and GvHD (8). These studies provide important clues about the pathophysiology of AIC following AHSCT since randomized clinical trials and pre-clinical modeling to understand mechanisms of AIC are lacking.

### Proposed Pathophysiology of AIC After AHSCT

**FIGURE 1:**
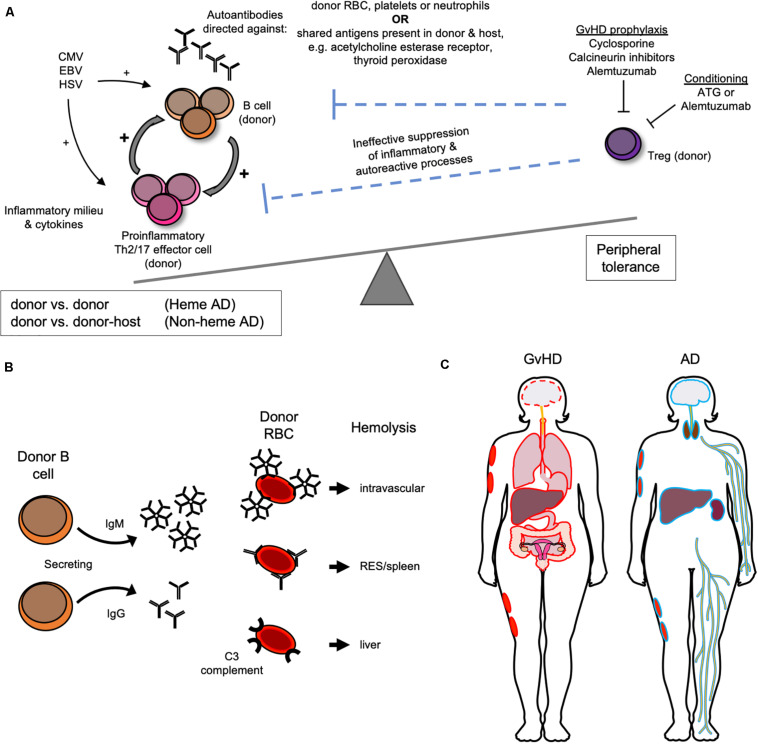
Biological and clinical features of autoimmune manifestations following AHSCT. **(A)** Proposed pathophysiology for the development of autoimmune manifestations after AHSCT as a result of donor T regulatory (T reg) cell impairment. **(B)** Donor immune reactions directed against donor red blood cell (RBC) antigens mediate autoimmune hemolytic anemia after AHSCT. **(C)** GvHD versus “autoimmune” non-hematologic tissue/organ targets outlined in red and blue, respectively.

As stated above, non-malignant disease and the use of unrelated donor grafts are most strongly (9) and consistently (10–12, 27, 28) associated with the development of AIC in children. The proposed pathophysiologic mechanism that could explain their combined role in the emergence of autoimmunity after HSCT is that AD may be driven by an impairment in peripheral immune tolerance due to the lack of functional T cells, in particular T regulatory cells (T_*regs*_) with resultant inability to suppress B cell expansion after HSCT (11), Figure 1A. In the post-transplant time frame when AICs typically emerge, the thymus has yet to recover from transplant related insults. Thus, peripheral tolerance mechanisms are likely dominant in keeping autoimmunity in check. Peripheral tolerance is mediated by T cells, which are expected to be preferentially eliminated in conditioning regimens used in unrelated donor HSCT that include ATG and alemtuzumab. Alemtuzumab targets CD52, which is expressed on T cells with greater density than other lymphocytes (34, 35), and can be particularly effective at inhibiting CD4 + T cell recovery compared to other T cell types (36). Both drugs have long lasting *in vivo* effects and would be expected to provide sustained T cell suppression in the post-HSCT period. T cell depletion is also commonly performed on haploidentical grafts, which too have been associated with greater propensity for AIC (33). Unregulated polyclonal B cell expansion would be more likely in the absence of T cell immunoregulatory signals combined with the anticipated pro-inflammatory viral stimuli commonly encountered in the immediate post-transplant period, such as CMV, EBV, and HSV infection or reactivation (23). In patients with cGvHD, another established risk factor for AIC after AHSCT (30), B cell alloantibody production is a common feature that stems from the inability of T_*regs*_ to dampen alloimmunity (37). Furthermore, cGvHD has been shown to respond to adoptive T_*reg*_ transfer in multiple pre-clinical and clinical studies (38, 39) and demonstrated the ability to prevent AIHA in animal models (17). Additionally, T_*reg*_ impairment is implicated in idiopathic AIHA (17, 40). Immunophenotyping of patients with AIC post AHSCT has confirmed low circulating CD4 and CD8 T cell numbers, low T_*reg*_ numbers (11, 26, 41), as well as Th2 skewing (13). The latter is a shared feature of idiopathic and AD associated AIHA (17, 40), and animal models of the former. Th17 polarization has also been implicated in the pathogenesis of idiopathic AIHA (40), but has yet to be confirmed in AHSCT-associated AD. Cyclosporine (CSA), the most common form of GvHD prophylaxis used in the multiple case series with higher AD rates presented above, would also be expected to have a greater impact on T rather than B cell subsets, in particular on IL-2 dependent expansion of T_*regs*_ (42). Of note, cyclosporine-, and calcineurin-based immunosuppression and incomplete lymphodepletion are associated with AICs after both solid organ transplantation (SOT) (41, 43–46) and non-malignancy HSCT and could point to shared biological mechanisms. Supporting this notion is the observation in the AHSCT AIC where withdrawal of CSA followed by anti-B cell directed therapy with rituximab or anti-CD38 resulted in clinical responses (11, 47). Finally, decades ago cyclosporine was shown to induce autologous GvHD-like reaction purportedly via disruption of peripheral tolerance (48).

While the pathophysiology of AICs is not fully understood, it does appear that AIHA is primarily driven by donor immune reactions against donor erythrocytes (9, 23) (Figure 1B). Donor chimerism was not uniformly reported in studies of AIC after AHST, but was usually full donor at the time of AIC diagnosis when reported (13, 23, 24, 31, 41), which implicates a donor against donor process. Thalassemia HSCT is characterized by higher AIC incidence, which could implicate prior transfusion and resultant alloimmunization playing a role in AIHA via the host versus donor response. Although if true, this would also be expected with other non-malignant indications, such as sickle cell disease, which have not been identified as risk factors for AIHA after AHSCT. Other AICs after AHSCT, ITP, and AIN, are also antibody mediated and evidence suggests that they too are donor against donor (12, 13, 41).

### Distinguishing AIHA From Major and Minor ABO Mismatch Hemolysis

While ABO mismatched AHSCT can be associated with delayed engraftment and other complications, it is often unavoidable in the HSCT setting (49) where HLA matching is prioritized over ABO and the two are not genetically linked (49). AIHA following AHSCT is distinct from ABO mismatch driven hemolysis, which can arise from either major or minor ABO mismatch between the donor and host (49). The ABO mismatch driven hemolysis can present with (1) immediate intravascular hemolysis mediated by host ABO antibodies directed against donor RBCs in the graft; (2) delayed hemolysis from residual host cells reacting to RBCs produced by the engrafted donor marrow, and (3) pure red cell aplasia (PRCA). Immediate intravascular hemolysis is more common when marrow is used as an HSCT source due to potential transfer of donor RBC in the stem cell graft, which can be minimized with RBC removal prior to graft infusion. Additionally, the timing of this clinical presentation readily distinguishes this alloimmune process from AIHA (49). Delayed hemolysis from residual host cells reacting to RBCs produced by the engrafted donor marrow typically occurs later in the post-HSCT period and mediates delayed engraftment commonly observed with ABO mismatched HSCT that typically occurs ∼5 weeks after graft infusion compared to ∼3 weeks that is routinely observed with ABO matched HSCT or peripheral blood stem cell grafts. Delayed hemolysis can present months after HSCT if full chimerism is not yet established and requires chimerism studies to distinguish it from AIHA. PRCA results from destruction of erythroid progenitors within the marrow by residual anti-donor antibodies. It may present in the same time frame as AIHA, within 6 months of HSCT, and have a similar clinical presentation despite the mechanisms between these entities being distinct, i.e., with ABO mismatch being host antibody driven and with AIHA being donor antibody driven (23) reactions against donor RBC.

### AIHA Diagnosis

Diagnosis of AIHA is established by performing a direct anti-globulin test (DAT), also called direct Coombs test, which detects *in vivo* coating of erythrocytes with antibodies (16). In the DAT, non-specific anti-human globulin will agglutinate RBCs coated by all antibody isotypes, IgA, IgM, IgG, etc. (16, 40) with agglutination more likely to be detected at warm testing temperatures when IgG antibody subtypes (IgG1, IgG2, IgG3, and IgG4), and/or C3 (complement) are present on the RBC and cold testing temperatures when IgM is bound. IgG mediates extravascular hemolysis via the reticuloendothelial system that is mainly splenic (Figure 1B). The pentameric antibody IgM is most efficient at fixing complement both in laboratory testing and *in vivo* and can cause significantly greater *in vivo* RBC destruction than other isotypes mainly via intravascular hemolysis. Meanwhile, C3-mediated hemolysis predominantly occurs in the liver. The majority of AIHA after AHSCT is warm type, followed by cold, then mixed (41). The type is important to ascertain because it can guide treatment, with cold agglutinin disease being less likely to respond to splenectomy since the RBC destruction would not be expected to occur in the reticuloendothelial system. If the DAT is positive, monospecific anti-IgG, anti-IgM, and anti-C3 antisera are used to further define the autoantibody. In cases of AIHA following AHSCT when monospecific testing was reported, IgG was commonly detected in combination with C3, with some cases of IgG, and IgM co-occurring. IgG when present was eluted to test for specificity. When looking at available studies, there was a suggestion that co-occurrence of warm and cold AIHA may have a more severe course (11), but no consistent pattern of severity of hemolysis or likelihood of response to treatment was clearly apparent for a particular type of antibody in post-AHSCT AIHA.

### Treatment and Prognosis of AIHA After AHSCT

Post-AHSCT AIHA is most commonly treated with intravenous immunoglobulin (IVIG) or steroids, rituximab monotherapy, plus a variety of other approaches. Only a third of the cases are steroid responsive. Rituximab has a reported ∼60–80% response rate. Other combinatorial immunosuppressive approaches have been described, including azathioprine, cyclosporine, 6-mercaptopurine, mycophenolate mofetil, danazol, cyclophosphamide and vincristine, bortezomib, alemtuzumab, sirolimus, and second stem cell infusion (9). A treatment strategy of reducing immunosuppression that is more heavily directed against T cells (i.e., CSA and calcineurin inhibitors) and instead using anti-B cell directed therapies in a subset of patients resulted in resolution of AIHA once T cell reconstitution was achieved (11). While prognosis appears to be marginally better in children than in adults, mortality in some cases did occur (9) and overall higher mortality in AHSCT recipients with AIHA compared to those without was reported (13). Also, AHSCT associated AIHA appears to be more treatment refractory (9) compared to non-AHSCT associated cases with the latter having ∼80% of response rate to corticosteroids within 3 weeks, splenectomy having a 70% response rate, and rituximab having a 60% response rate. Several recalcitrant cases of post-AHSCT AIC, including AIHA, have been recently reported to respond to daratumumab (47, 50, 51), which targets CD38 that is expressed on plasmablasts and plasma cells.

### Other Hematologic Autoimmune Manifestations

Immune thrombocytopenic purpura after ASHCT appears to have slightly higher incidence in children than adult recipients (9, 52), but is quite rare in both, limiting understanding of its pathophysiology and prognostication. Idiopathic (53) and post-AHSCT ITP (54) have been associated with T_*reg*_ dysfunction. In both clinical settings, ITP diagnosis is that of exclusion. Antibodies against platelets were not consistently obtained across the reported case series and reviews of ITP occurring after AHSCT, but when testing was reported, ∼75% of the clinically determined cases were associated with detectable direct and indirect anti-platelet antibody (12, 28). The antigenic specificity of the antibody was even less frequently reported, but when available appeared to be directed against similar thrombocyte antigens as non-HSCT associated ITP, i.e., platelet membrane glycoproteins IIb-IIIa or Ib-IX. As already described for AIC in general, donor chimerism was most often full donor at the time of diagnosis, confirming that the post-AHSCT ITP is frequently autoimmune, i.e., donor against donor. Passive transfer of donor ITP has been described in the adult AHSCT literature (31, 52), but not in the pediatric setting. For post-AHSCT ITP and Evans syndrome, systemic corticosteroids and IVIG were the typical first line treatment, with a majority of the patients eventually receiving multiple lines of therapy, including rituximab, which resulted in a few complete responses (30, 31), daratumumab (47, 51), vincristine, cyclophosphamide, azathioprine, 6-mercaptopurine, alemtuzumab, plasma exchange, stem cell boost, splenectomy, rapamycin, romiplostim, and eltrombopag (28). It appears that ITP after AHSCT is often chronic, recalcitrant to treatment, and can result in mortality (15, 28, 31, 55).

Autoimmune neutropenia after AHSCT is also an antibody driven process, although in most AIC studies confirmatory testing of direct or indirect anti-neutrophil antibodies was not reported, and when reported was infrequently positive (12, 14, 48). Similar treatment approaches for AIN have been reported as for AIHA, ITP, and Evans syndrome, but again, given the rarity of this AHSCT complication, prognostication is not appropriate. Acquired hemophilia, i.e., development of factor VIII inhibitors, has been reported in the setting of autologous HSCT for AD (15, 20, 48, 56, 57), but not in the setting of adult or pediatric AHSCT. Thrombotic microangiopathic manifestations following AHSCT as a result of high ADAMTS 13 inhibitor levels have been described (8) albeit not in children.

## Non-Hematologic “Auto” Immune Diseases After AHSCT

Non-hematologic manifestations after AHSCT that are potentially autoimmune in mechanism, involving the thyroid, central and peripheral nervous systems, integument, liver, and kidney are far less common in children than in adults and have not been extensively described or reviewed in the setting of AHSCT (Figure 1C). Whether these conditions are autoimmune or alloimmune is an ongoing research question because immune effectors are of donor origin but are directed against targets that are common to the donor and host, e.g., acetylcholine esterase receptor, thyroid peroxidase, etc. These conditions are antibody mediated, which is also the case outside of the AHSCT setting when they are truly autoimmune. Such ADs have also been reported in the setting of dysregulated immunity associated with immunosuppression after SOT where the autoimmune reaction is that of host against host. In contrast, GvHD is a common, well-recognized, and better described immune driven complication of AHSCT that is mediated by donor immunity against non-hematopoietic host organs and tissues. Donor against host directed antibodies are implicated in GvHD pathophysiology. While myasthenia gravis (MG) and peripheral neuropathies are not formally part of cGvHD diagnostic criteria, they are considered “other” or “associated features” of cGvHD when present concomitant with classical GvHD manifestations and are observed in the setting of cGvHD. Whether non-hematologic thyroid, peripheral and central nervous system (PNS and CNS) manifestations and vitiligo should be considered “other GvHD” or referred to as autoimmune is not clear at this time. Since in the adult HSCT literature they are most often described as autoimmune, in this review they will be referred to as such for consistency.

### Autoimmune Thyroid Disease

Thyroid ADs after AHSCT, mainly Hashimoto thyroiditis and Graves’ disease, are mediated by antibodies against thyroid antigens and have been described primarily in the setting of autologous and allogenic HSCT for AD in adults (8, 15, 22, 48, 58–60), and AHSCT for non-malignant indications in children (10, 55, 61–63). For the reported series in children, common features were T cell depletion (graft *ex vivo*, ATG or alemtuzumab peri-transplant), non-malignant indication (all cases described here), lack of TBI (in all cases summarized here), and an incidence of 1–25%. The highest Autoimmune Thyroid Disease (AITD) incidence was described in a cohort of 24 boys that received matched sibling or unrelated donor AHSCT after alemtuzumab conditioning (10), with 5 cases of Graves’ disease and one case of Hashimoto thyroiditis, which represents an unusually high rate. Of note, non-AHSCT AITD has also been associated with alemtuzumab treatment in multiple sclerosis (62). Multiple reports discussed in this review of autoimmune hematologic manifestations in children did not observe AITD and three pediatric case series that described AITD had also reported on hematologic AD occurring at higher rates than AITD (55). In the pediatric series that reported the highest AITD rate, above average incidence of AIC of 20% was also observed, as was the rate of CNS and PNS manifestations with 8% and 4%, respectively. Thus, this series appears to have had unique features resulting in significantly higher rates of AITD, an otherwise rare complication of AHSCT. In both children and adults, AITD diagnosis after AHSCT was established with appropriate antibody detection and treatment approaches were supportive, i.e., thyroid suppression for hyperthyroidism and replacement hypothyroidism. Unlike hematologic AD, there were no deaths attributable to AITD in the pediatric studies. In adult AHSCT reports of AITD, possible transmission of donor autoantibodies in the graft has been reported (8, 48, 64–66) while in children this etiology does not appear to play a role. Interestingly, one study noted that recovery of CD4 T cell counts was coincidental with onset of AITD, similar to the setting of immune restoration in patients with HIV in whom AITD has also been reported (62). In many of the pediatric post-AHSCT AITD cases reviewed here, onset was typically later than that of AICs, perhaps suggestive that immune recovery, albeit dysfunctional since it resulted in a manifestation of autoimmunity, may play a role in the emergence of AITD in the post-AHSCT setting.

Intriguingly, another autoimmune mediated endocrinopathy, type 1 diabetes mellitus, has not been described in the pediatric studies reviewed here for hematological and non-hematological AD after AHSCT or otherwise, while insulin resistance after AHSCT is commonly described in adults and children after AHSCT (67–69).

### Neurological Autoimmune and Graft-Versus-Host Disease Manifestations After AHSCT in Children

Neurological manifestations of autoimmunity after AHSCT and cGvHD have been reported to affect central and peripheral nervous systems (8, 70). Neurological manifestations are not included in the definitive cGvHD diagnostic criteria, but nonetheless are considered “associated” with cGvHD when involving the PNS: peripheral nerve, including Guillain-Barre syndrome (GBS), neuromuscular junction, i.e., MG, and muscle. Of the latter, myositis and polymyositis are deemed the only “distinctive” neurological manifestations of cGvHD (70); however, these entities are not described in this review due to paucity of reports on these manifestations in pediatric AHSCT setting. MG and peripheral neuropathies are considered “other” GvHD or “associated with GvHD” in the presence of classical cGvHD manifestations in other organs. For CNS manifestations to be regarded as “definitively” cGvHD, they have to occur with classic cGvHD manifestations in other organs, other causes have to be excluded, and presence of imaging, CSF, and biopsy proven evidence of alloreactivity and proven response to immunosuppression are necessary (70). Notably, most antibody driven neurological entities after ASHCT manifest in the setting of full donor chimerism, hence the cGvHD or autoimmune processes again arises.

#### PNS Manifestations

Guillain-Barre syndrome is a rare complication of AHSCT in children and adults (8, 48, 71, 72), and in the latter more likely in allogenic than autologous HSCT (72). We found 10 reported cases of pediatric GBS after HSCT in the literature (10, 71, 73–75). Of those, 8 were in the setting of HSCT for a malignant indication and 2 for CGD (10). In the former, 2 cases were reported following autologous HSCT (71, 74), and 2 were associated with infection, bacterial sepsis, and parainfluenza 1. No evidence of GvHD had been described in all 10 cases, although 4 occurred within the immediate post-transplant window, hence potential association with GvHD would not be evaluable (73, 75). In adult recipients, GBS has been associated with GvHD (70) and with infection/reactivation of CMV, HSV, and HHV6 (8, 71, 76, 77) as well as antecedent infections (70). A strong association between GBS in the first week after AHSCT and the use of antecedent high dose Ara-C was observed in two reports involving 4 children (73, 75), and resulted in 3 fatalities (75). The remaining patients responded to treatment with systemic corticosteroids, IVIG, plasmapheresis, and rituximab.

Two cases of MG after HSCT have been reported in children, both in the setting of non-malignant indication and mismatched sibling AHSCT (78, 79). In both instances, MG was generalized (non-focal), was associated with cGvHD and manifested as immunosuppression was being tapered, with one patient having also experienced acute GvHD and the other engraftment syndrome. Acetylcholine receptor antibodies were present in both cases. One patient was treated with pyridostigmine, atropine, AZA, thymectomy and plasmapheresis, and was eventually responsive to thalidomide (78). The other patient was found to be initially ANA positive prior to the development of MG manifestations and presented with severe generalized MG that required intubation and eventually resolved after treatment, which consisted of MMF, IVIG, methylprednisolone, pyridostigmine, cyclosporine, plasma exchange, and finally a course of rituximab. Notably, outside of the HSCT setting, MG is quite rare in children and when it occurs in pre-pubertal setting is often ocular and remains so without generalization. In such settings it is also less likely to be antibody positive and has a favorable prognosis, including reported spontaneous remissions (80). In the adult HSCT literature, 23 cases of MG have been reported after allogeneic and autologous HSCT (8, 48, 70), with a majority of the patients having acetylcholine receptor antibodies and few with musculoskeletal receptor antibodies. The majority were associated with cGvHD and most presented upon discontinuation or tapering of cGvHD immunosuppression. Treatment was similar to idiopathic MG, i.e., pyridostigmine, acetylcholine esterase inhibitors, and immunosuppression. In contrast to MG in adults in the non-HSCT setting there was no observed association with thymoma (8). Of note, MG in the setting of immunosuppression (ATG or alemtuzumab) after renal transplantation has been reported (81).

Myositis and polymyositis although rare after AHSCT are associated with cGvHD (70), but have not been well described in children.

#### CNS Manifestations

CNS immune manifestations that are cGvHD related have been rarely reported in adult HSCT literature and can be ascribed to cerebrovascular, stroke-like presentations, encephalopathy with resultant seizures, and demyelination processes (70, 82–84). Isolated cases of immune CNS disease after HSCT have been reported in children (85–88), and only one of these presented as transverse myelitis (TM) that was not associated with GvHD, but was associated with AIC and GBS (10), while several cases of TM after HSCT have been reported in adults (8, 89). Isolated optic neuritis has also been described in the adult AHSCT literature (70), but has not been reported in the pediatric AHSCT setting. A case of CNS granulomatous angiitis/vasculitis was described in an 18-year old recipient of mismatched unrelated graft, in association with weaning of immunosuppression for resolving acute GI GvHD. The patient was found to have generalized CNS atrophy on MRI, which was obtained due to progressive spasticity and seizures. The patient’s cognitive dysfunction worsened further to progressive encephalopathy with concurrent skin cGvHD onset that manifested 5 months after HSCT (87). Interestingly, short tandem repeat (STR) analysis of a micro dissected section of her inflamed arteriole confirmed that the lymphocytic infiltrate was of donor chimerism. The patient improved following very high doses of steroids, stabilized, but eventually lost her graft and died 2 years after HSCT. Similarly, in adult patients with CNS-GvHD, infiltrating lymphocytes were of donor origin (70, 83) suggesting that CNS-GvHD can be appropriately classified as a GvHD manifestation. Two case reports have described diffuse white matter lesions in children with GvHD and a case of cortical atrophy associated with cGvHD (85, 86). While CNS immune manifestations after HSCT in children are extremely rare, they are primarily associated with GvHD and tapering of immunosuppression and likely represent alloimmune rather than autoimmune processes.

### Skin Autoimmune GvHD-Associated Manifestations After AHSCT

Vitiligo is a rare manifestation in the AHSCT setting, which is mostly observed with concomitant acute or chronic skin GvHD. A total of 8 pediatric vitiligo cases after HSCT have been described in the literature in several case series combining adult and pediatric patients (55, 90–93). Cathcart et al. described one pediatric case of extensive vitiligo developing 4 years after mismatched sibling T cell depleted HSCT for malignancy, interestingly without concomitant chronic GvHD, albeit with a history of resolved acute skin and liver GvHD (92). In the same series, nine adult cases of post-HSCT vitiligo were described. All had been transplanted for a malignant indication and were associated with acute or chronic GvHD. Another case series reporting on vitiligo after AHSCT for malignant indications included a pediatric patient with extensive vitiligo after AHSCT for ALL associated with skin and GI GvHD (91). Sanli et al. described six cases that were observed in a single transplant center in 421 consecutive patients (93). One of these 6 cases occurred in a 19-year-old who developed vitiligo 6 months after matched sibling HSCT for CML, and which was associated with liver cGvHD, alopecia areata, and subsequently oral and lichenoid skin GvHD. Finally, five additional cases of vitiligo in children were reported, one in association with lichenoid skin cGvHD after AHSCT for aplastic anemia (94) and four in the setting of peripheral blood stem cell grafts for hemoglobinopathy (55) without an association with GvHD or other AD. Vitiligo has been associated with autoimmunity outside the HSCT setting (95) and can be mediated by antibodies directed against melanocytes (55, 96), and thus could represent an autoimmune process. Nevertheless, alloimmunity cannot be excluded in this clinical entity as half of the reported pediatric cases of vitiligo after AHSCT were associated with concomitant classic skin cGvHD.

### Other Rare Autoimmune Manifestations After HSCT

Whether autoimmune-like hepatitis (AIH) occurring after HSCT truly represents an autoimmune manifestation versus drug-associated hepatitis, i.e., by cyclosporine, is difficult to ascertain since only two pediatric cases (55, 97) and one adult case have been reported (98). The liver biopsy in all three cases showed portal eosinophilia and plasma cell infiltration, with chimerism of the lymphocytic infiltrate demonstrated to be of donor origin in the adult patient. In one pediatric case after AHSCT for a metabolic disorder there was no concurrent or prior history of GvHD (55) and in the other AIH was associated with GvHD (97), as was the case reported in the adult. All three cases were steroid responsive. In the adult patient, azathioprine was used in combination with steroids and in one pediatric case ursodiol was combined with steroid (97). This was the only patient that had been on cyclosporine prior to AIH diagnosis, upon which it was discontinued. It was not clear from the description of the case whether the other pediatric patient had been on cyclosporine after HSCT.

Whether the kidney is a target of autoimmunity after AHSCT or a manifestation of GvHD-associated alloimmunity is not clear. Reports of membranous nephropathy and minimal change disease have been described in the post-HSCT setting, most commonly observed in association with cGvHD and particularly upon weaning of immunosuppression (99). However, a few cases have occurred in the autologous HSCT setting without GvHD (100), with two such reports in children (101, 102). The pathophysiology of these renal manifestations appears to be mediated by antigen-antibody complexes deposited in the glomerular subendothelium as a result of either kidney antigens being targeted or indirect injury from deposition of the complexes targeting antigens exogenous to the kidney (99). The pediatric cases of immune-mediated nephropathy after HSCT had been reportedly treatment responsive to systemic immunosuppression with corticosteroids, which are also used outside of the HSCT setting for these clinical entities.

Rheumatologic diseases possibly autoimmune in etiology have been described after autologous and allogeneic HSCT, including rheumatoid arthritis, psoriatic arthritis, spondyloarthropathy, vasculitis, and antiphospholipid antibody syndrome, more commonly after AHSCT for autoimmune indications (8, 15, 48). In one report, two young adult patients had been described as having possible autoimmune arthritis (103). A 24-year-old man who underwent AHSCT for a T cell lymphoma and antecedent history of arthritis (concomitant ANA titers negative) developed acute symmetrical polyarthritis involving the proximal interphalangeal (PIP) and metacarpophalangeal (MCP) joints, wrists, and knees 1 month after discontinuation of post-HSCT immunosuppression. This was associated with a high ANA titer. The patient experienced spontaneous resolution of symptoms 6 months later. The other patient was a 2l year-old woman who received matched sibling HSCT for AML, with resolved acute skin GvHD skin and ongoing lung cGvHD. She developed bilateral shoulder and unilateral hip and knee pain, with arthrocentesis finding of coagulase-negative Staphylococcus. Despite appropriate antibiotic treatment, synovitis now involving bilateral knees and several PIP joints persisted and was then treated with anti-inflammatory medications (not specifically stated) and intra-articular injection of the knee with a corticosteroid, which resulted in complete resolution of the symptoms. Whether the arthritis was immune- or infection-driven in the latter case is not entirely clear. Otherwise, autoimmune arthritities have been almost exclusively described in the setting of HSCT for autoimmunity (15, 22), which indicates that a predisposition toward autoimmunity in combination with the transplant factors likely plays a pathophysiological role in such manifestations.

## Future Directions and Summary

Identification of risk factors for autoimmune manifestations in children and adults undergoing ASHCT as well as comprehensive diagnostic characterization of these rare cases are imperative to advance the understanding of the biological mechanisms behind these complex set of conditions. Many AD manifestations remain poorly understood due to lack of prospective studies and pre-clinical models. The former would be difficult to implement due to rarity of these complications, but subsets of patients have been identified to have higher inherent risks for developing AD (8, 9), which could facilitate such efforts. Such studies should include the collection of clinically well annotated samples of blood and tissue. Innovative study designs adapted to rare entities and development of novel minimally invasive biological sampling techniques suitable for pediatric patients are imperative to move this area of research forward. Emerging diagnostic approaches could provide further mechanistic insights into pathophysiology of manifestations. For example, single cell sequencing approaches are now able to capture TCR diversity, and as analytical methods advance valuable insights into antigenic targets, i.e., TCR specificity, may be identified (104). Patient outcomes could be improved by selection of targeted treatments (if targets are known), which would potentially be more effective and less toxic than general immunosuppression (105). For example, unregulated B cell expansion had been implicated as a potential mechanism of AD and the use of anti-B cell agents has demonstrated clinical efficacy in steroid refractory cases (28, 48). T_*reg*_ dysfunction is a common feature of GvHD (106) and AD after AHSCT and perhaps *in vivo* T_*reg*_ expansion is a strategy that could be attempted in the setting of AD after ASHCT. Janus tyrosine kinase (JAK) inhibitors were developed for ADs outside of the AHSCT setting (107) and have elicited clinical responses in patients with steroid refractory GvHD (108), which indicates they may also be efficacious in AD after AHSCT. In conclusion, AD likely stems from T and B cell dysfunction in the context of pro-inflammatory post-AHSCT milieu in which immunoregulatory processes are impaired. As risk factors for the development of AD after ASHCT are better characterized and the underlying biology is better understood, patients and families can be appropriately advised about the risks, changes to the existing BMT platforms can be implemented, and therapeutic targeting of underlying biology can be explored.

## Disclosure

The views expressed here are of the authors and do not represent views of the NIH or the US government.

## Author Contributions

NB and SP contributed to the conception of the article. NB wrote the manuscript. SP contributed to the manuscript revision. All authors contributed to the article and approved the submitted version.

## Conflict of Interest

The authors declare that the research was conducted in the absence of any commercial or financial relationships that could be construed as a potential conflict of interest.
